# Technologies for Cognitive Training and Cognitive Rehabilitation for People With Mild Cognitive Impairment and Dementia. A Systematic Review

**DOI:** 10.3389/fpsyg.2020.00648

**Published:** 2020-04-09

**Authors:** Eider Irazoki, Leslie María Contreras-Somoza, José Miguel Toribio-Guzmán, Cristina Jenaro-Río, Henriëtte van der Roest, Manuel A. Franco-Martín

**Affiliations:** ^1^Faculty of Psychology, University of Salamanca, Salamanca, Spain; ^2^Department of Research and Development, Iberian Research Psycho-Sciences Institute, INTRAS Foundation, Zamora, Spain; ^3^Department on Aging, Netherlands Institute of Mental Health and Addiction (Trimbos-Institute), Utrecht, Netherlands; ^4^Department of Psychiatry, University Rio Hortega Hospital, Valladolid, Spain; ^5^Zamora Hospital, Zamora, Spain

**Keywords:** cognitive training, cognitive rehabilitation, software, cognitive impairment, dementia, systematic review

## Abstract

Due to the growing number of older adults with cognitive impairment, it is essential to delay the onset and progression of cognitive decline and promote a healthy lifestyle. The rapid growth of technology has considerably advanced the field of computerized cognitive interventions. Consequently, traditional cognitive interventions are being adapted and new multimedia systems are being developed to encourage health and independent living in old age. The primary objective of this review was to identify cognitive stimulation, training and rehabilitation programs aimed at older people with mild cognitive impairment (MCI) and dementia. PsycINFO, Medline, CINAHL, Web of Science, PubMed, and CORDIS databases were searched from January 2008 to August 2018. Two researchers reviewed the potential studies individually for eligibility. Studies of computerized cognitive interventions for people with dementia and cognitive impairment were included if they clearly described objectives, users and functioning. A systematic review of the studies was carried out, providing a qualitative synthesis of the features and study characteristics of each software. Nineteen studies met the inclusion criteria, and 11 different cognitive stimulation, training, and rehabilitation programs were identified. The studies found on cognitive intervention software indicate the existence of various technological programs for people with MCI and dementia. On the overall, the programs were aimed at people with different clinical conditions, able to create specific treatments and personalized training, optimized for portable devices, and user-friendly. However, the selected programs differ from each other in terms of objectives, usage mode and characteristics, even if they were used for the same purposes. Therefore, the information obtained in the review may be relevant to distinguish between programs and select the one that best suits each user. Thus, more information about the features and context of use is needed as well as more clinical studies to be able to compare among computerized cognitive programs.

## Introduction

Despite the advances in treatments of chronic diseases related to old age, dementia is considered one of the most significant public health challenges (Nemeth et al., 2017). It is estimated that 74.7 million people around the world will be living with dementia by the year 2030 (Alzheimer's Disease International., 2015). In Spain alone, currently, over 800.000 people are affected by dementia (Alzheimer-Europe, 2013). This number will rise, since it is estimated that in 2050, Spain will be one of the oldest countries in the world, with 40% of the population being over 60 years by then (United Nations, 2015).

Mild cognitive impairment (MCI), an intermediate stage between healthy aging and dementia, is also a common condition in older people (Petersen et al., 2001). It is estimated that 10–20% of the population over 65 are affected by MCI (Petersen, 2011). MCI can be amnesic (aMCI), non-amnesic (naMCI) and the impairment could affect a single cognitive domain (sdMCI) or multiple domains (mdMCI) (Petersen et al., 2014). Alzheimer's Disease has been frequently associated with aMCI (Lange et al., 2018), while naMCI may increase the risk for other dementias such as frontotemporal dementia and dementia with Lewy bodies (Ferman et al., 2013).

Since there is no cure for dementia, attempts have been made to identify factors that may delay the onset and slow progression of cognitive decline in people with cognitive impairment. Similarly, in order to hamper the course of dementia for as long as possible and to enable people to age in place, many different types of psychosocial approaches that aim to improve and maintain cognitive ability have been developed in the last decades (Klimova and Maresova, 2017; Wei et al., 2020).

The concepts of cognitive reserve and neuroplasticity have gained attention as potential factors for delaying cognitive decline (Soldan et al., 2017). Cognitive reserve has been described as the structural and dynamic capacity of the brain to cope with changes associated with natural aging or injuries. Due to this pre-existing cognitive processing approach, people with higher cognitive reserve deal better with pathologies, atrophies, or injuries (Stern, 2012). Following this reasoning, a recent review demonstrates that cognitive reserve might be linked to dementia prevalence and specific cognitive domain performance (Lavrencic et al., 2018). Conversely, neuroplasticity is the brain's ability to generate morphological changes in response to an environmental stimulus (Shaffer, 2016). Due to this ability, our brain can adjust and compensate for cognitive alterations by strengthening existing connections or creating new ones. Brain's cognitive reserve and plasticity are influenced across the lifespan by several factors such as genetics, educational level, occupation, socioeconomic factors, physical health, lifestyle, and mental activity (Sampedro-Piquero and Begega, 2017).

The limited efficacy of pharmacological therapies and the neuronal plasticity of our brain are the main reason for the growing interest in non-pharmacological treatments (Takeda et al., 2012). For improvement of cognitive functioning in people with cognitive impairment and dementia, three types of non-pharmacological cognitive interventions have been developed over time. Cognitive stimulation refers to a wide variety of non-specific exercises focused on cognitive and social functioning reinforcement (Clare et al., 2003). Discussions, reminiscence therapy (Irazoki et al., 2017) and reality orientation (apart of other features) are examples of stimulation techniques that are mostly administered in a group setting. Cognitive stimulation was found to have a positive effect on cognition of people with mild to moderate dementia (Streater et al., 2016). Cognitive training aims to maintain or improve a particular aspect of cognitive functioning (e.g., memory or attention) through structured and guided practice carried out individually or in a group (Bahar-Fuchs et al., 2019). The difficulty level of activities can be adapted to individual functioning. Regarding efficacy, it has been demonstrated that cognitive training can improve the general cognitive functioning of people with mild dementia (Tsantali et al., 2017). Finally, cognitive rehabilitation is an individualized intervention explicitly focusing on a person's needs (Clare et al., 2013). The emphasis is on improving or maintaining cognitive abilities related to everyday task performance, compensating impairments and supporting and enhance independent living (NCC for Mental Health., 2007). It is considered as one of the most effective interventions since it has shown to be able to slow down the progression of cognitive decline in people with dementia (Amieva et al., 2016). All intervention types must be executed under the control of a professional therapist.

Many traditional cognitive interventions have been adapted for use on current technological devices such as smartphones, tablets and computers, as they are considered a cost-effective alternative as compared to conventional cognitive interventions (Meiland et al., 2017).

Cognitive training, stimulation, and rehabilitation provided via digital devices are promising strategies for maintaining the cognitive function of healthy older adults and people with MCI (Zhang et al., 2019). Computerized cognitive interventions are not only useful for improving cognition, memory, and attention but also have a positive influence on the psychosocial functioning of older adults with MCI (Hill et al., 2017). Similarly, it was demonstrated that computerized cognitive training's beneficial effects remained on both short-term and long-term in people with preserved cognitive function (Ten Brinke et al., 2018).

The computerized cognitive intervention has several advantages over traditional techniques (García-Casal et al., 2016). Zokaei et al. (2017) identified that training tasks are useful because they (a) can be directed to a specific cognitive function (e.g., memory, attention); (b) can be continually adjusted based on the participant's performance; (c) can be designed to be highly immersive and enjoyable; (d) provide instant quantitative feedback; and (e) are actively accessible on portable digital devices. Indeed, in the computer approaches for improving cognitive function is possible to apply a mix up of cognitive stimulation, cognitive training and cognitive rehabilitation with the same devices or computer (González-Palau et al., 2014).

Consequently, the field of computerized cognitive interventions is growing steadily, as well as the research projects related to these technologies. The recently published studies focus on the effectiveness of computer-based cognitive intervention for people with dementia (Gates et al., 2019a,b). Still, little is known about the individual characteristics of each computerized program. So far, the computerized programs for improving the cognitive function have been considered as part of the same therapy without taking into account the significant differences between each other. The present review aims to identify and compare computerized cognitive stimulation, training, and rehabilitation software for older adults with MCI and dementia. Specifically, it is intended to determine the characteristics and the differences and similarities between the diverse computerized programs, as all programs are similar but not identical even though they are used for the same purposes.

## Methods

### Materials

Details for this systematic review were registered on PROSPERO (CRD42019117531)^1^. The study was performed considering PRISMA (Preferred Reporting Items for Systematic Reviews and Meta-Analyses) guidelines for bibliographic reviews (Urrútia and Bonfill, 2010) and included randomized controlled trials, study protocols, and pilot studies regarding cognitive stimulation, cognitive training and cognitive rehabilitation software for older adults with dementia and MCI.

### Procedure

PROSPERO (https://www.crd.york.ac.uk/prospero/) was searched to ensure that no other systematic review had been registered previously on this topic. No such study was identified.

The databases PsycINFO, Medline, CINAHL, Web of Science and PubMed were searched from January 1, 2008, to August 31, 2018. The following search terms were used in combination: (“comput^*^” OR “computer software”) AND (“brain training” OR “cognitive training” OR “memory training” OR “cognitive rehabilitation”) AND (“Alzheimer” OR “frontotemporal dementia” OR “vascular dementia” OR “cognitive impairment”). Additionally, we searched the Commission database of EU-funded research and innovation projects (CORDIS) for “computer-based cognitive rehabilitation” and “computer-based software for cognitive impairment.” The searches were filtered by health domain of application and project collection. We also searched for gray literature using Google Scholar looking for “computer-based software” AND “cognitive training and cognitive rehabilitation” AND “dementia”. The research was limited to the years 2008–2018. Additionally, the reference lists of available studies were screened for further potentially eligible articles.

Studies were included if they described: (1) software for people over 60 years; (2) computer-based cognitive stimulation, cognitive training and cognitive rehabilitation programs; (3) technologies aimed at people with Alzheimer's Disease, frontotemporal, or vascular dementia, or people with mild cognitive impairment (amnestic, non-amnestic and multiple domain); (4) technologies with clear descriptions of the objective, users and functioning; (5) were published between 2008 and 2018 and (6) written in English or Spanish.

Exclusion criteria were: (1) technologies exclusively aimed at healthy people; (2) technologies aimed at people with other types of dementia as described above (e.g., Lewy bodies, Pick's disease) or other clinical populations; (3) games, assistive technology, robots and virtual reality; (4) programs that do not require a therapist; (5) systematic reviews, meta-analysis and editorials.

### Procedure Study Selection

**Figure 1:**
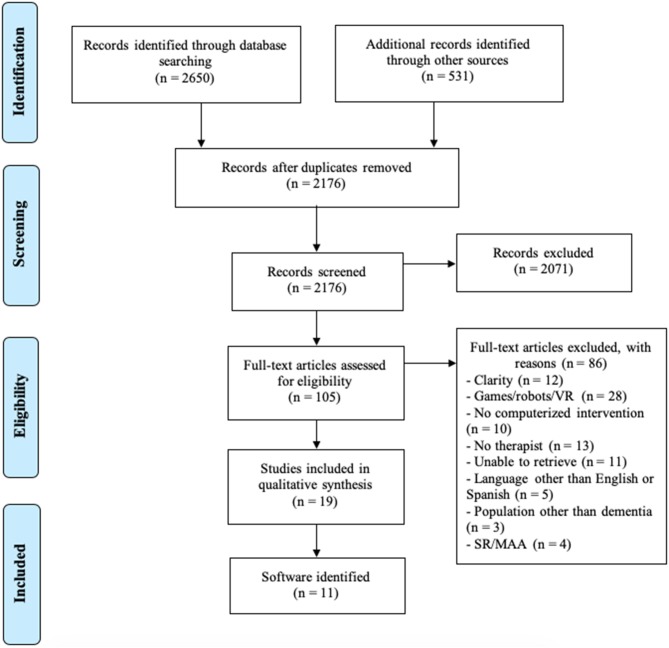
Flow chart of the search strategy.

Two researchers independently reviewed the titles and abstracts of identified studies for eligibility and screened the full text of potentially available studies (E.I. and LM. C-S.). The researchers compared their reviews and agreed upon inclusion by consensus. In case of disagreement, a third reviewer (JM. T-G.) was consulted. No metric of inter-rater reliability was kept. Figure 1 summarizes the process of selecting studies.

### Data Extraction

Data collection included the individual characteristics of all computerized programs. Initial data extraction was based on the information available in the selected studies. Subsequently, every technology was looked upon on the web, as a secondary source for additional information. The features to analyze were chosen considering the basic requirements for technology to have clinical usefulness (Franco-Martín et al., 2002) and are shown in Table 1. Once the identification of the articles was completed, we analyzed the characteristics of these studies considering the number and type of studies, participant demographic characteristics, intervention details, and the main results.

**Table 1:** The individual characteristics for analysis of computerized programs.

**Variables**	**Description**
Intervention type	Cognitive stimulation Cognitive training Cognitive rehabilitation
Usefulness	Stimulated cognitive functions
Flexibility with each user	Program capacity to personalize and adapt the content to the specific cognitive profile of the end-users
Disease flexibility	Competency to be used by people with different clinical conditions (mild, moderate, or severe degree of dementia)
Accessibility	Remote applicability Internet connection Application type (native or web app)
Portability	Device type
User-friendliness	Input/output device
Content	Exercises or tasks Progress report Additional features

### Data Analysis

As a result of the different features of the technologies and methodological differences of the studies, we provide a qualitative synthesis of the results considering Cochrane guidelines for data synthesis and analysis (Ryan, 2013). First, we provide a brief description of the feature of the software. Secondly, we summaries the characteristics of the selected studies and mention the studies found concerning these computerized programs.

## Results

### Characteristics of Computerized Programs

A total of 2,650 articles and 531 projects were obtained from the search. 2,176 studies remained after the exclusion of duplicates, and the titles and abstracts of identified papers were first reviewed for elimination. The identified documents were classified according to inclusion and exclusion criteria, leaving 105 potential articles to include in the review. Finally, 19 studies were selected to perform the analysis (Figure 1).

The identified cognitive stimulation, training, and rehabilitation software for people with MCI and dementia are shown in Table 2. A total of 11 computerized computer programs were identified, precisely four programs for cognitive rehabilitation (Brainer, GRADIOR, NeuronUp, ComCog), six for cognitive training (Captain's Log, Cogmed, CogniFit, CogniPlus, COGPACK, SOCIABLE) and one for cognitive stimulation (FesKits). It is necessary to take into account that in many cases, the computer programs mix up the different cognitive approaches and consequently, they were classified considering the primary strategy used.

**Table 2:** Characteristic of identified computerized cognitive programs.

**Program and Website**	**Type of intervention**	**Target group**	**Device**	**Targeted cognitive functions**	**Input device**	**Output device**	**Application type**	**Remote application**	**Internet connection required**	**Flexibility**	**Content**	**Progress report**	**Additional features**
**Brainer** www.brainer.it	CR	Neurological disorders	PC, tablet^*^	VP, AP, attention, R&W, language, calculus, logic and deduction, memory, SMS	Mouse, touch screen^*^	NA	Web Native^*^	Yes^*^	Yes^*^	Yes	78 exercises	Yes^*^	NA
**Captain's log** www.braintrain.com/captains-log-mindpower-builder	CT	CI, TBI, MI, ADHD^*^	PC^*^	Memory, attention, perception, reasoning, planning, judgment, EF^*^	Mouse^*^	Headset^*^	Native^*^	Yes^*^	Yes^*^	Yes^*^	2000 exercises^*^	Yes^*^	Entertaining Games Assessment battery
**Cogmed**® www.cogmed.com	CT	ADD, LD, TBI, CI, stroke	PC, iPad/tablet^*^	WM	Mouse^*^	Headset	Web Native	Yes^*^	Yes	Yes^*^	25 training session	Yes^*^	Cogmed Coach
**CogniFit** www.cognifit.com	CT	HOP, ADHD, depression, PD, stroke, dyscalculia, dyslexia, insomnia, fibromyalgia	PC, iPad/ tablet, smartphone	Attention, memory, EF, perception, reasoning coordination	Keyboard, mouse	Headset^*^	Web	Yes	Yes	Yes	33 tasks	Yes^*^	Assessment tools
**CogniPlus** www.schuhfried.com	CT	BD, ADHD, MCI^*^	PC^*^	Attention, memory, SP, planning, visuomotor skills	Mouse, keyboard, Schuhfrieds Basic response panel^*^	Headset^*^	Native^*^	NA	No^*^	Yes	15 tasks^*^	Yes^*^	Physical exercises
**COGPACK** www.cogpack.com	CT	PD, neurological disorders^*^	PC	Visuomotor skills, logic, language, orientation, comprehension, memory, problem-solving^*^	Keyboard, mouse or touch screen	NA	Native ^*^	NA	No^*^	NA	537 task sets^*^	Yes^*^	NA
**FesKits** www.feskits.com	CS	HOP, stroke, TBI, tumors, dementia, MS, PD, DS, ID schizophrenia	PC, laptop	Attention, memory concentration, EF, perception, recognition, language, calculus, spatial and temporal orientation	Keyboard, mouse	Headset^*^	Web	Yes^*^	Yes^*^	Yes	> 5,000 exercises^*^	Yes^*^	NA
**GRADIOR** www.intras.es	CR	HOP, NDD, MI, NPD, BD, cerebral palsy, dementia	Touchscreen computer	Attention, perception, memory, orientation, calculation, language, EF, reasoning	Mouse (optional)	Headset^*^	Native	Yes	Yes^*^	Yes	>12,500 exercises	Yes^*^	Assessment tools
**NeuronUp** www.neuronup.com	CR	AD, MS, PD, stroke, ADHD, dementia, MI, NDevD, ID^*^	NA	Memory, attention, gnosis, EF, praxis, language, social cognition and visuospatial skills^*^	NA	NA	Web^*^	Yes^*^	Yes^*^	Yes^*^	> 6,000 activities^*^	Yes^*^	Serious Games and additional resources
**ComCog** https://home.neofect.com/blog/tag/rapael-comcog	CR	AD, Dementia, Stroke, TBI^*^	Tablet^*^	Attention and memory	NA	NA	Web^*^	NA	Yes^*^	Yes^*^	> 20 exercises^*^	Yes^*^	NA
**SOCIABLE** www.cognitivetraining.eu	CT	MCI, mAD, HOP	Multi-touch surfaces (tablet, PC)	Memory, orientation, attention, EF, language, praxis, reasoning	Multitouch surfaces^*^	NA	NA	Yes^*^	Yes^*^	Yes^*^	25 exercises^*^	Yes^*^	Social interaction tasks

* *, information obtained in the web; AD, Alzheimer Disease; ADD, Attention Deficit Disorder; ADHD, Attention Deficit Hyperactivity Disorders; ADL, Activities of Daily Living; AP, Auditory Perception; BD, Brain Damage; CI, Cognitive Impairment; CR, Cognitive Rehabilitation; CS, Cognitive Stimulation; CT, Cognitive Training; DS, Down Syndrome; EF, Executive Function; HOP, Healthy Older People; ID, Intellectual disabilities; LD, Learning Disorders; mAD, Mild Alzheimer Disease; MCI, Mild Cognitive Impairment; MI, Mental Illness; MS, Multiple Sclerosis; NA, Not Available; NDD, Neurodegenerative Disorders; NDevD, Neurodevelopmental Disorders; NPD, Neuropsychiatric Disorders; PC, Personal Computer; PD, Parkinson's Disease; PS, Processing Speed; R&W, Read & Write; SMS, Sensory Motor Skills; SP, Spatial Processing; TBI, Traumatic Brain Injury; VP, Visual Perception; WM, Working Memory*.

Overall, the identified technologies were flexible tools for each end user's cognitive profile. The programs allowed therapists to create tailored treatments and to adjust the difficulty level of exercises to every user (Brainer, Captain's Log, CogniFit, COGPACK, FesKits, GRADIOR, NeuronUp, ComCog), even automatically (Cogmed, CogniPlus, ComCog). Programs were usable for other clinical groups in addition to people with MCI and dementia. People with disorders such as dyslexia, insomnia, multiple sclerosis, Parkinson's disease and brain damage could also benefit from the majority of identified cognitive software. Moreover, four technologies could be used by healthy people as a way to prevent cognitive decline (CogniFit, FesKits, GRADIOR, SOCIABLE). Regarding usefulness, most of the identified programs targeted multiple cognitive domains, while just one was specifically designed to enhance working memory (Cogmed).

Six web-type technologies (Brainer, Cogmed, CogniFit, FesKits, NeuronUp, ComCog) and four native applications (Captain's Log, CogniPlus, COGPACK, GRADIOR) were found (designed for specific mobile platforms). One technological program was available in both native and web-based applications (Cogmed), and another computerized program offered a web-based application for users and a native application for professionals (Brainer). Internet connection was required for most of the programs, while only two native apps worked off-line (COGPACK, CogniPlus). Furthermore, eight computerized programs enabled remote use (Brainer, Captain's Log, Cogmed, CogniFit, FesKits, GRADIOR, NeuronUp, SOCIABLE), whereas, for the rest of the programs, this was not specified.

Most programs were optimized for both personal computers and laptops (Brainer, Captain's Log, Cogmed, CogniFit, CogniPlus, COGPACK, FesKits, GRADIOR, SOCIABLE), computers with touch screen (GRADIOR), and iPad, tablet or smartphones (Brainer, Cogmed, CogniFit, ComCog, SOCIABLE). Keyboard (CogniFit, CogniPlus, COGPACK, FesKits) and mouse (Brainer, Captain's Log, Cogmed, CogniFit, CogniPlus, FesKits, CogniFit) were the most common input devices. However, mouse use was set as optional (COGPACK, GRADIOR, SOCIABLE). One technology program can function with a standard computer keyboard or with SCHUHFRIED's Basic response panel, a particularly suitable keyboard for individuals with restricted hand movement (CogniPlus). Additionally, most of the programs required the use of headsets (Captain's Log, Cogmed, CogniFit, CogniPlus, FesKits, GRADIOR).

The content of the programs varied in terms of the number of tasks and exercises. Some programs contained 15–25 activities, and others had over 2,000 exercises. It was also found that the 11 programs generated progress reports of users' cognitive performance. Another significant feature was that three of the software included neurocognitive assessment tools (Captain‘s Log, CogniFit, GRADIOR). Finally, one of the software combined cognitive tasks with physical exercises (CogniPlus), and another could also be used for individualized and group cognitive training as well as to reinforce social interactions (SOCIABLE).

Table 3 summarizes the differences and similarities between computerized cognitive programs according to the characteristics considered most appropriate to make such technology as useful as possible for both users (in this case, older people with MCI and dementia) and therapists.

**Table 3:** Differences and similarities between the reviewed tools.

	**Multiple target group**	**Touchscreen device**	**Multiple target function**	**Accessories**	**Headset**	**Web application**	**Remote application**	**Internet connection**	**Flexibility**	**Progress report**	**Additional features**
Brainer	Yes	Yes	Yes	Yes	NA	Yes	Yes	Yes	Yes	Yes	Yes
Captain's Log	Yes	No	Yes	Yes	Yes	No	Yes	Yes	Yes	Yes	Yes
Cogmed	Yes	Yes	No	Yes	Yes	Yes	Yes	Yes	Yes	Yes	Yes
CogniFit	Yes	Yes	Yes	Yes	Yes	Yes	Yes	Yes	Yes	Yes	Yes
CogniPlus	Yes	No	Yes	Yes	Yes	No	NA	NA	Yes	Yes	Yes
COGPACK	Yes	No	Yes	No	NA	No	NA	NA	NA	Yes	NA
FesKits	Yes	No	Yes	Yes	Yes	Yes	Yes	Yes	Yes	Yes	NA
GRADIOR	Yes	Yes	Yes	No	Yes	No	Yes	Yes	Yes	Yes	Yes
NeuronUp	Yes	NA	Yes	NA	NA	Yes	Yes	Yes	Yes	Yes	Yes
ComCog	Yes	Yes	Yes	NA	NA	Yes	NA	Yes	Yes	Yes	NA
SOCIABLE	Yes	Yes	Yes	No	NA	NA	Yes	Yes	Yes	Yes	Yes

*NA, Not Available*.

### Characteristics of the Selected Studies

Table 4 summarizes the selected studies classified by the identified computerized programs for the current systematic review. We found 19 studies in which 11 digital cognitive training programs for older people with cognitive impairment and dementia were mentioned. The selected papers consisted of ten RCTs (Gaitán et al., 2012; Zaccarelli et al., 2013; Fiatarone Singh et al., 2014; Barban et al., 2015; Cavallo et al., 2016; Hyer et al., 2016; Suo et al., 2016; Bahar-Fuchs et al., 2017; Hagovská et al., 2017; Cavallo and Angilletta, 2018), five pre-post studies (Gigler et al., 2013; González-Palau et al., 2014; Hwang et al., 2015; Vermeij et al., 2017; Mendoza Laiz et al., 2018), two studies with repeated measures design (Eckroth-Bucher and Siberski, 2009; Vermeij et al., 2016), one pilot study (Danassi, 2015), and one study protocol for an RCT (Vanova et al., 2018).

**Table 4:** Details of the studies that support the use of the identified computerized cognitive programs.

**Name**	**References**	**Study design**	**Participants**	**Intervention**	**Duration**	**Primary outcomes**	**Main results**
Brainer	Cavallo et al., 2016	RCT	Early stage of AD EG: 76.5 ± 2.88 CG: 76.33 ± 3.83 29 M, 31 F	Individual EG: CT (*n* = 40) CG: leisure activities (*n* = 40)	F: 3 t/w D: 30 m/s; 12w	Cognition, memory, semantic knowledge, language, visuospatial abilities, EF	- EG significant effects on short-term memory; WM; oriented memory; language comprehension and EF - Improvements remained at 6 months follow up
	Cavallo and Angilletta, 2018	RCT	Early stage of AD EG: 76.5 ± 2.88 CG: 76.33 ± 3.83 29 M, 31 F	Individual EG: CT (*n* = 40) CG: leisure activities (*n* = 40)	F: 3 t/w D: 30 m/s; 12 w	Cognition, memory, semantic knowledge, language, visuospatial abilities, EF	- Significant effects on short-term memory; WM; oriented memory immediate and delayed; language comprehension; EF
Captain's Log	Eckroth-Bucher and Siberski, 2009	Repeated measures	NI, MI and MoI 78.6 ± 8.43 5 M, 27 F	Individual EG: CT+P&P (*n* = 17) CG:—(*n* = 20)	F: 2 t/w D: 45 m/s; 6 w	Cognition, logical memory	- MI and MoI groups show significant improvements in DRS and logical memory—Improvement maintained after 8 weeks
Cogmed®	Hyer et al., 2016	RCT	aMCI and naMCI EG 75.1 ± 7.4 CG 75.2 ± 7.8 32 M, 36 F	Individual EG: CT adapted (*n* = 34) CG: CT no-adapted (*n* = 34)	F: - D: 40 m/s; 5–7 w	WM, IADL, subjective memory complaints	- Significant changes in non-verbal WM and subjective memory complains - IADL improved for EG at the follow up (12 weeks)
	Vermeij et al., 2016	Repeated measures	HOA, aMCI and a-md MCI 67.8 ± 2.4 23 M, 12 F	Individual CT (n = 47)	F: 5 t/w D: 45 m/s; 5 w	WM	- HOA perform better than people with MCI - Both groups improved on the Digit Span and Spatial Span and maintained at follow-up (3 months)
	Vermeij et al., 2017	Pre-post	HOA, aMCI and a-md MCI 67.8 ± 2.4 23 M, 12 F	Individual CT (*n* = 47)	F: 5 t/w D: 45 m/s; 5 w	WM	- MCI group improved WM performance after training
CogniFit	Bahar-Fuchs et al., 2017	RCT	MCI, NPS and NPS+MCI 74.6 ± 6.8 24 M, 20 F	Individual EG: personalized (*n* = 21) CG: pre-determined (*n* =23)	F: 3 days/week; 2 session/day D: 20–30 m/s; 8–12 w	Cognition	- MrNPS performed better than MrNPS + MCI in cognition; delayed memory; learning and memory; and non-memory composite
	Gigler et al., 2013	Pre-post	HOA and aMCI 89.33 ± 16.33 5 M, 13 F	Individual CT (n = 18)	F: 2 t/w D: 20–30 m/s; 8–10 w	Cognition, everyday task, QoL, IADL	- Higher scores for HOA in an auditory memory span, visual memory and WM
CogniPlus	Hagovská et al., 2017	RCT	MCI Group A: 67.8 ± 6.5 Group B: 68.2 ± 4.2 29 M, 31 F	Individual Group A: CT (*n* = 30) Group B: TCT (*n* = 30)	F: 2 t/w D: 30 m/s; 10 w	Functional activities, QoL, cognition, attention	- Group A performed better on QoL, cognition and attention - No differences were found on functional activities
COGPACK	Fiatarone Singh et al., 2014	RCT	MCI 70.1 ± 6.7 -	Individual TG1: CT (*n* = 24) TG2: PRT (*n* = 22) TG3: CT+PRT (*n* = 27) CG: videos, stretching; toning (*n* = 27)	F: 2 t/w D: 60–100 m/s; 26 w	Cognition, IADL, EF, memory and attention	- TG2 significantly improved cognition at 6 months and executive function across 18 months. TG1 only attenuated the decline in Memory Domain at 6 months
	Suo et al., 2016	RCT	MCI 70.1 ± 6.7 32 M, 68 F	Individual TG1: CT (n = 24) TG2: PRT (n = 22) TG3: CT+PRT (n = 27) CG: videos, stretching; toning (n = 27)	F: 2 t/w D: 90 m/s; 26 w	Cognition, IADL, EF, memory and attention	- Significant results for TG2 on cognition - TG1 improved results on overall memory performance
FesKits	Gaitán et al., 2012	RCT	a-md MCI and AD G1: 76 ± 6.61 G2: 74.87 ± 4.89 19 M, 20 F	Individual G1: CBCT+TCT (n = 37) G2: TCT (n = 23)	F: 2-3 t/w D: 60 min; 12w	Attention, PS, memory, EF, praxis, gnosis and cognition	- A nearly significant interaction for EF in G1. Results remained at 12 months follow up
GRADIOR	González-Palau et al., 2014	Pre-post	aMCI, a-md MCI and HOA 73.43 ± 7.51 10 M, 40 F	Individual CT and Physical training (*n* = 50)	F: 3 t/w D: 40 m/s; 12 w	Cognition, mood	- Improvement of cognitive function and verbal and episodic memory in both groups; and decreased symptoms of depression
	Vanova et al., 2018	Study protocol	aMCI and mD -	Individual G1: CT (*n* = 100) G2: PSS (*n* = 100) G3: CT+PSS (*n* = 100) G4: TAU (*n* = 100)	F: 3–4 t/w D: 30 m/s; 12 months	Cognition, QoL, ADL, mood, Patient-carer relationship	-
NeuronUp	Mendoza Laiz et al., 2018	Pre-post	MCI 68.18 ± 4.28 14 M, 18 F	Individual NFT and WMT (*n* = 32)	F: 1 t/w D: 80 m/s; 5 w	Attention, intellectual process, memory, spoken language and visuospatial ability	- G1 improved on VP; spatial orientation; receptive speech; expressive speech; memory; picture recognition; concepts - G2 improved on picture recognition; concepts
ComCog	Hwang et al., 2015	Pre-post	AD 14 M, 21 F	Individual CT (*n* = 35)	F: 5 t/w D: 30 m/s; 4 w	Cognition	- A significant decrease in recognition and increase on orientation, registration and recall
SOCIABLE	Barban et al., 2015	RCT	HE, MCI, mAD T1: 74 ± 2.92 T2: 73.93 ± 2.6 129 M, 172 F	Individual or in group T1: pb/CT+RT/Rest (*n* = 149) T2: Rest/pb/CT+RT (*n*= 152)	F: 2 t/w D: 60 m/s; 12w	Memory and EF	- Significant effects on memory and in HE groups on EF -The effects remained at 6 months follow up on MCI and HE groups
	Danassi, 2015	Pilot study	HE, MCI, mAD -	Individual or in group CT (*n* = 315)	F: 2 t/w D: 3 months	Cognition, affection, functional abilities	- Significant improvement on cognition and functionality; depression unchanged -Improvements remained at 3 months follow up
	Zaccarelli et al., 2013	RCT	HE, aMCI and mAD -	Individual or in group EG: CT (*n* = 174) CG: - (*n* = 174)	F: 2 s/w D: 60 m/s 12w	Cognition, memory, praxis, EF, attention, language	- Significant results on cognition; memory and EF; constructional praxis and language

*AD, Alzheimer Disease; ADL, Activities of Daily Living; aMCI, amnestic Mild Cognitive Impairment; a-md MCI, amnestic-multiple domain Mild Cognitive Impairment; CBCT, Computer-Based Cognitive Training; CT, Cognitive Training; CG, Control Group; D, Duration; DRS, Dementia Rating Scale; EG, Experimental Group; EF, Executive Function; F, Frequency; G, Group; HE, Healthy Elderly; HOA, Healthy Older Adults; IADL, Instrumental Activity of Daily Living; m/s, Min/session; mAD, Mild Alzheimer's Disease; MCI, Mild Cognitive Impairment; mD, mild Dementia; MI, Mild Impairment; MoI, Moderate Impairment; MrNPS, Mood-Related Neuropsychiatric Symptoms; na-MCI, non-amnestic Mild Cognitive Impairment; NFT, Neurofeedback Training Sessions; NI, No Impairment; NPS, Neuropsychiatric Symptoms; pb-CT, Process-Based Cognitive Training; PRT, Progressive Resistance Training; PS, Processing Speed; PSS, Psychosocial Stimulation; P&P, Paper & Pencil; QoL, Quality of Life, RCT= Randomized Controlled Trial; RT, Reminiscence Therapy; T, Treatment; TAU, Treatment As Usual; TCT, Traditional Cognitive Training; TG, Treatment Group; t/w, times/week; VP, Visual Perception; W, Weeks; WM, Working Memory; WMT, Working Memory Training*.

The study participants were between 60 and 91 years and people with MCI (mean age: 73.5 ± 5.3), an early stage of AD (mean age: 76.4 ± 3.35), moderate cognitive impairment (mean age: 78.6 ± 8.43) or with Alzheimer's Disease (mean age: 76.2 ± 1.1), and healthy older people (mean age: 72.2 ± 2.9). The number of individuals included in each study varied, ranging from 17 to 348. In general, more women participated in the studies, and no differences between arms were found.

Regarding the intervention characteristics, the duration of the interventions varied considerably between 4 and 26 weeks. There was also substantial heterogeneity in both the number and length of the sessions. On average, the interventions were provided 2–3 times per week for 46 min.

### Scientific Studies for Computerized Programs

Analyzing the effectiveness of cognitive intervention software was not the objective of the review because there are currently papers doing it (Gates et al., 2019a,b). Nevertheless, it was considered essential to mention the characteristics of the population and the principal findings of the included studies.

#### Cognitive Stimulation Program

A 12-months intervention with the cognitive stimulation software FesKits was evaluated in an RCT in comparison to a traditional cognitive training program (Gaitán et al., 2012). The study was carried out with people with MCI and Alzheimer's Disease and showed that the group receiving both traditional and computer-based cognitive training improved in the performance of executive function tasks.

#### Cognitive Training Programs

Captain's Log is a computerized program for cognitive training. No other details about this cognitive software were described in the identified studies. Captain's Log was part of an Integrated Cognitive Stimulation and Training Program intervention, in which a combination of other stimulation techniques was used (Eckroth-Bucher and Siberski, 2009). A repeated measures experimental study was carried out with participants with mild and moderate cognitive impairment and healthy older adults. The results showed that people with mild and moderate impairment receiving a combination of stimulation techniques enhanced the logical memory domain and that these improvements remained 8 weeks after the intervention.

Three studies were identified regarding the use of the computer-based cognitive training program Cogmed (Hyer et al., 2016; Vermeij et al., 2016, 2017). Hyer et al. (2016) conducted an RCT that examined the effectiveness of Cogmed in older adults with MCI. The study found that non-verbal working memory and subjective memory complaints of participants improved after 5–7 weeks of cognitive training. In a repeated measure design study, Cogmed was used to analyze the transfer effects of working memory (Vermeij et al., 2016) and the prefrontal activation after training in a pre-post study (Vermeij et al., 2017). In both studies, people with MCI and healthy older adults were included. The main results showed improvements in working memory tasks and maintenance of these effects 3 months post-intervention.

CogniFit cognitive training software was evaluated in an RCT (Bahar-Fuchs et al., 2017) in people with MCI and people with mood-related neuropsychiatric symptoms (MrNPS). The study showed that people with MrNPS performed overall better than participants with MCI in global cognitive ability. Another study aimed to explore the potential of CogniFit in people with MCI and healthy adults (Gigler et al., 2013). The pre-post study found that participants in the cognitive training condition improved on global cognition and memory after the intervention.

CogniPlus is computerized software for cognitive training. Its effectiveness was compared in an RCT study to a traditional group-based program in older adults with MCI (Hagovská et al., 2017). The study showed that the group receiving computerized training performed better on cognition, attention and had a better quality of life.

Two studies were found regarding the use of the cognitive training software COGPACK (Fiatarone Singh et al., 2014; Suo et al., 2016). Fiatarone Singh et al. (2014) carried out an RCT regarding the Study of Mental and Resistance Training (SMART) with people with MCI. The study showed that the group receiving computerized training improved memory function after 6 months of training while the group receiving resistance training showed significant improvements in cognition and executive functions as compared to control conditions. Similarly, the RCT conducted by Suo et al. (2016) aimed to examine structural and functional brain changes after cognitive training and resistance training in people with MCI. The study showed significant improvements in cognition for the resistance training group and better memory performance for the computerized training group.

Two RCT studies were found in which the cognitive training program SOCIABLE was evaluated (Zaccarelli et al., 2013; Barban et al., 2015). Barban et al. (2015) examined the effects in combination with group Reminiscence Therapy in people with MCI, mild Alzheimer's Disease and healthy subjects. The results showed that people with MCI and mild Alzheimer's Disease maintained cognitive function after the intervention. Furthermore, the study of Zaccarelli et al. (2013) found that cognition, memory, executive functions, language and praxis were improved after the intervention with this program. A pilot study was also carried out with SOCIABLE in four European countries (Danassi, 2015). This study involved participants with MCI, mild Alzheimer's Disease and healthy older adults and the results showed positive effects for people with MCI and healthy older adults in cognition and functional abilities while mood state did not change.

#### Cognitive Rehabilitation Programs

The cognitive rehabilitation program Brainer was evaluated in two RCT studies in people with early-stage Alzheimer's Disease (Cavallo et al., 2016; Cavallo and Angilletta, 2018). The studies found that the intervention influenced working memory, language comprehension and executive functions positively and that these effects remained 6 months after the intervention but decreased after 12 months.

The cognitive rehabilitation software GRADIOR was part of The Long Lasting Memories European project that aimed to validate an integrated technology platform combining cognitive exercises with physical activity (González-Palau et al., 2014). A pre-post study was carried out with people with MCI and healthy subjects, and the results showed significant improvements in global cognitive function and symptoms of depression. Also, a study protocol regarding the efficacy of GRADIOR was identified (Vanova et al., 2018). The study protocol described an RCT with an envisaged total of 400 people with MCI and mild dementia to determine the effectiveness of the cognitive rehabilitation program GRADIOR and the ICT platform ehcoBUTLER, separately and in combined treatment.

NeuronUp is a program for cognitive rehabilitation. Its effectiveness was evaluated in a pre-post study that aimed to analyze the improvements in the neurological profile of people with MCI and Alzheimer's Disease (Mendoza Laiz et al., 2018). The study found an increase in picture recognition and concepts in both groups.

Hwang et al. (2015) conducted a pre-post study to examined the effects of the cognitive rehabilitation program ComCog on the global cognition of people with Alzheimer's Disease and concluded that participants performed better on orientation and information registration while no improvements in recognition were observed.

## Discussion

This systematic review discloses the state of the art on cognitive intervention software providing cognitive stimulation, training, or rehabilitation for older adults with MCI and dementia. The review aimed to check the characteristics of computer-based cognitive programs and the differences and similarities between the existing software, avoiding considering that all computer programs working for cognitive improvement are identical. We focused the study on the software used in regular computers, considering that they are more used than others. Probably in the future, tablets, smartphones, or other devices can be used more often, but currently, the usability of computers is higher than the other technologies for people with dementia (Góngora Alonso et al., 2019). We identified 19 studies that used 11 different cognitive software programs for the treatment of people with MCI and dementia independently if they were used for cognitive stimulation, training, or rehabilitation.

Like traditional cognitive interventions (Lobbia et al., 2018), most identified computerized programs were aimed to improve multiple cognitive domains, where memory and attention were the most stimulated cognitive functions. The review identified computerized programs with standardized training sessions as well as software that enables to create new treatments, define training goals and customize training parameters such as difficulty level, session duration and session frequency. Programs with standardized training sessions are unable to modify or adapt treatments to the cognitive profile of end-users (e.g., Cogmed, Brainer, CogniPlus, FesKits). This non-flexibility of programs is a significant disadvantage since the training is the same for everyone, even if the difficulty level of the exercise changes. It was also found that some programs can automatically propose exercises of the most appropriate cognitive difficulty level.

In this study, software aimed at people with Alzheimer's Disease were mainly included since it is the most common type of dementia (Garre-Olmo, 2018). Vascular dementia and frontotemporal dementia were also considered because of their high prevalence (Hogan et al., 2016; Wolters and Arfan Ikram, 2019). In general, cognitive intervention programs are not explicitly aimed at people with dementia, but also to other clinical conditions. None of the identified technologies was designed expressly for dementia alone, as many targeted a broad range of disorders causing cognitive impairment. The fact that programs are suitable for many clinical conditions can be seen as an indicator of the strength and flexibility of the programs. Even more, in most cases, they have been designed to improve the cognitive functions independently of the origin of the problem. It means that they consider mainly the cognitive function and less the special features of every disease.

This review found two native apps that did not require an internet connection (CogniPlus and COGPACK) and eight programs that allowed the remote use of cognitive software (Brainer, Captain's Log, Cogmed, CogniFit, FesKits, GRADIOR, NeuronUp, SOCIABLE). Online platforms force users to have an internet connection, a requirement that native applications might not have. The need for internet connection may be inconvenient, especially for older people who do not have access to the internet at home or in nearby facilities. This is the case of people living in further rural areas without access to the internet. However, programs working through the internet enable remote applicability, which may be a potential approach to improve the availability of treatments of people who live in rural areas and experience difficulties in accessing health care services. Furthermore, online platforms allow users to work on different devices, participation in treatment programs regardless of location, and even facilitate data sharing. Applications that do not require internet connection cannot be used remotely, the therapist cannot supervise treatment, and the settings/levels cannot be automatically tailored.

All computerized cognitive training (CCT) were available on conventional (portable) digital devices, which facilitates the uptake and implementation of the intervention. Similarly, it was found that the interaction between programs and end-users slightly differ within programs. Half of the tools were developed for use with a touch screen or an adapted keyboard, which makes it easy for people with computer illiteracy to use them because the similarity with TV is high. Almost all computer-optimized technologies can also be used with a mouse, although mouse usage requires a higher cognitive level than touch screens or other devices. As keyboard and mouse control can be a barrier for older people, the need for designing user-friendly programs that do not require lots of accessories is logical. Therefore, programs optimized for touch screen devices could encourage people with dementia to participate in computer-based interventions (Joddrell and Astell, 2016). Older people may experience fear of using computers due to a lack of experience or familiarity (Góngora Alonso et al., 2019). Training people with dementia in the use of technologies and providing support during the interventions might be effective strategies to promote the use of technological devices (Meiland et al., 2017).

All programs generated reports of treatment results. This feature provides the opportunity to monitor improvement, performance and evolution of each user. In this sense, computer programs facilitate data management and making an adequate follow-up of the intervention. However, it was not possible to check the differences among them in the accuracy of the reports.

Another characteristic of computerized programs is that they are designed to be enjoyable and fun. The identified digital software contained a wide variety of exercises that stimulate engagement and avoid repetition. Moreover, some software even combined entertaining games and motivating video games to enhance the user's performance in the intervention. It has been proven that brain games or cognitively stimulating leisure activities may also help to prevent or delay the effects of aging (Yates et al., 2016). However, most of these cognitive enhancement activities are easily accessible and regularly performed for entertainment with no need for any professional monitoring. Therefore, these types of brain games are designed with a completely different purpose and should not be considered as treatments for people with cognitive decline and dementia.

According to this systematic review and the identified software, all features might have advantages and disadvantages. Also, depending on the context of use and the characteristics of each person, one computerized program might be more suitable than another. In our opinion, web-based software working through an internet connection would facilitate the implementation of the intervention since it could be applied in any device with an internet connection and would facilitate the remote applicability. In terms of content, we believe that the more cognitive exercises the program contains, the easier it will be to maintain users motivated. It would also be considered favorable if the software contained evaluation tools and progress reports that would help the therapists to personalize and monitor the intervention. Besides, touch screen computers may be most suitable for use with older people with MCI and dementia (Lim et al., 2013). While computers may be less portable than other devices such as tablets, the size of the screen may seem more appropriate for use by older people who may feel more comfortable with this type of equipment. It is also recommended to designed simple intervention programs and that do not require too many accessories (Van der Roest et al., 2017).

Analyses of the characteristics of these programs showed that the identified strengths and weaknesses of cognitive intervention software are in line with previous studies comparing online neuropsychological rehabilitation platforms (Guerrero-Pertíñez and García-Linares, 2015). The authors concluded that online platforms should consider the possibility of comparing results between people with similar characteristics, create personalized exercises or task as well as making computers more accessible for people with sensory-motor deficits. Similarly, interventions should be as simple as possible and more tailored to the needs of people with dementia (Van der Roest et al., 2017).

Although it was not one of the main objectives of the review, the studies selected for the review were examined regarding the methodology used for proving their usefulness. Half of the studies were RCTs, with relatively small sample sizes. Additionally, five pre-post studies, two studies with a repeated measures design, a pilot study and study protocol were identified. The size of the study samples varied between 17 and 348 participants, though most of them included fewer cases than required to offer robust evidence. Considering the number and the study type of each software, SOCIABLE was the one that was most correctly evaluated, with two RCTs and acceptable sample sizes.

Additionally, almost half of the studies were conducted with two intervention groups, CCT for the experimental group and traditional cognitive training, leisure activities, stretching and toning exercises, or pre-determined computerized training tasks for the control group. Only two programs were compared against conventional cognitive intervention (CogniPlus and FesKits). These studies found nearly significant results on quality of life, cognition, attention (Hagovská et al., 2017) and executive functions (Gaitán et al., 2012). Three studies provide combined treatments in the intervention condition, and eight studies did not consider any control group. Consequently, we cannot find strong evidence in all these studies. It is essential to conduct more RCTs on the effectiveness of the computerized cognitive intervention and long-term follow-ups to reach more robust conclusions (Gates et al., 2019a,b).

Some limitations should be considered concerning the conclusions of this review. First, studies published longer than 10 years ago were not considered, since they probably studied outdated technologies or programs not functioning anymore. Secondly, computer-applied software were only explored, excluding researches using other devices such as a smartphone or tablet. In general, the usability of those devices in dementia is lower than the computer and currently, it is advisable to use a computer with big touch screens (Góngora Alonso et al., 2019). Thirdly, the effectiveness of the computerized programs was not analyzed since the aim of the study was not to establish the usefulness of the software. Several recent systematic reviews concerning the efficacy of cognitive computer software are available (García-Casal et al., 2016; Gates et al., 2019a,b; Hu et al., 2019; Zhang et al., 2019). Furthermore, due to the lack of cost-effectiveness information, it was not possible to compare the programs with this respect. Besides, the specific outcome measures used in the studies were not taken into account and the risk of bias of the studies was not assessed since the objective of this review was not to analyze the potential efficacy of these multimedia resources. Finally, the identified cognitive intervention software were similar but not the same in terms of characteristics such as objectives and function, which made it challenging to compare the programs.

It also should be noted that the information obtained from the selected studies was considered as the primary source. However, almost the studies offered only an elementary description of cognitive training programs, and in some cases, the characteristics of the technologies were not even reported. It is recommended to add a more comprehensive description of the computer programs since they are an essential part (intervention) of the studies. Given the limited information available in the identified studies, it was necessary to search for information on the website of each technology.

One of the strengths of this systematic review is that it offers an understanding of the different cognitive intervention software for people with MCI and dementia. The study also provides details of the main characteristics and requirements of each technological program, which allows comparing among different software. In this way, it becomes clear that computerized cognitive intervention programs are similar but not the same. Moreover, although studying the effectiveness of computer programs was not one of our objectives, the review provides an overview of the studies related to each program, as well as the results obtained in terms of computerized intervention effects on aspects such as cognition, mood and quality of life. The information collected in this review may also be relevant for health care providers who want to implement a computerized cognitive intervention in the clinical setting. However, it is necessary to clarify that these technological programs are only supportive tools for the assessment and treatment of the cognitive functions, but in no case, replace the role of the therapist in the intervention. Computer-based cognitive interventions should always be monitored by a professional who supervises emotional, psychosocial and behavioral aspects. However, the identified computerized interventions could facilitate the therapist's work in terms of efficacy in the planning, design, and management of cognitive treatments.

Finally, the literature shows a great variety of computer programs aimed at the field of dementia and cognitive impairment, as well as the effects of interventions in the area of research (Butler et al., 2018). However, a future search could be oriented to assess the actual use of these technological tools in clinical practice as part of a treatment or intervention provided to older people with cognitive impairment. In other words, it would be useful to check whether these computerized programs are available to users if they achieve the objectives for which they were designed or whether they remain in research projects.

## Conclusion

Eleven computerized programs to improve the cognitive functioning of older adults with dementia or MCI were identified in this systematic review. The scientific evidence on these programs was reported in 19 studies with various study designs. The analysis shows that computer programs differ from each other in terms of objectives, features and functions. This variety of programs allows professionals and end-users to choose the one that suits best with their interests and goals as not all people have the same needs, and not all programs are optimal for all people. However, web-based programs containing numerous exercises of different cognitive functions, without many accessories and applied to computers with large touch screens, might be the most appropriate cognitive programs for people with MCI and dementia. Besides, computer programs seem to be a promising strategy for enhancing the cognitive function of older people as they are more accessible (Maldonado, 2016) and cost-effective in comparison to traditional cognitive interventions (Gooding et al., 2015). Investing in more clinical studies and complying with better user-standards might be useful approaches to create meaningful and practical technology and to make more robust comparisons between different cognitive software. It is also necessary to describe the main features of these computerized programs in more detail as there may be studies that do not sufficiently specify the computer program used in the investigation. Finally, more information on the context of use is essential to improve the knowledge on how to use CCT effectively to delay the progression of cognitive impairment in people with MCI and dementia.

## Author Contributions

EI and MF-M contributed to the conception and design of the study. EI and LC-S performed the data collection supervised by JT-G. EI wrote the first draft of the manuscript. CJ-R, MF-M, and HR revised the manuscript critically for relevant intellectual content. MF-M and EI revised the last version of the manuscript. All authors contributed to manuscript revision, read and approved the submitted version.

### Conflict of Interest

EI, LC-S, and JT-G are directly linked to INTRAS Foundation, which has been the main developer and promoter of the GRADIOR software for cognitive rehabilitation. The remaining authors declare that the research was conducted in the absence of any commercial or financial relationships that could be construed as a potential conflict of interest.

## Footnotes

^1^It can be accessed at http://www.crd.york.ac.uk/PROSPERO/display_record.php?ID=CRD42019117531
